# Supplementary material for: Glucokinase Inactivation Ameliorates Lipid Accumulation and Exerts Favorable Effects on Lipid Metabolism in Hepatocytes
Source: Int J Mol Sci. 2023 Feb 21;24(5):4315. doi: 10.3390/ijms24054315 (PMC10002408; doi:10.3390/ijms24054315)
Supplement: Supplementary file 1 [file ijms-24-04315-s001.zip › ijms-2209403-supplementary.pdf]

Supplemental

Supplementary Table

Table S1. The full names and corresponding abbreviations of lipids.

Abbreviation	Full name of lipids
ACar	Acylcarnitine
BMP	Bismonoacylglycerophosphate
CE	Cholesteryl ester
Cer	ceramides
Cer-ADS	Ceramide alpha-hydroxy fatty acid-dihydrosphingosine
Cer-AP	Ceramide alpha-hydroxy fatty acid-phytospingosine
Cer-AS	Ceramide alpha-hydroxy fatty acid-sphingosine
Cer-BDS	Ceramide beta-hydroxy fatty acid-dihydrosphingosine
Cer-BS	Ceramide beta-hydroxy fatty acid-sphingosine
Cer-EODS	Ceramide Esterified omega-hydroxy fatty acid-dihydrosphingosine
Cer-EOS	Ceramide Esterified omega-hydroxy fatty acid-sphingosine
Cer-NDS	Ceramide non-hydroxyfatty acid-dihydrosphingosine
Cer-NP	Ceramide non-hydroxyfatty acid-phytospingosine
Cer-NS	Ceramide non-hydroxyfatty acid-sphingosine
CL	Cardiolipin
DAG	Diacylglycerol
DGDG	Digalactosyldiacylglycerol
DGTS	Diacylglyceryl trimethylhomoserine
FA	Free fatty acid
FAHFA	Fatty acid ester of hydroxyl fatty acid
GlcADG	glucuronosyldiacylglycerol
GM3	Ganglioside
HBMP	Hemibismonoacylglycerophosphate
HexCer-AP	Hexosylceramide alpha-hydroxy fatty acid-phytospingosine
HexCer-NDS	Hexosylceramide non-hydroxyfatty acid-dihydrosphingosine
HexCer-NS	Hexosylceramide non-hydroxyfatty acid-sphingosine
LDGTS	Lysodiacylglyceryl trimethylhomoserine
LPA	Lysophosphatidic acid
LPC	Lysophophatidylcholine
LPE	Lysophosphatidylethanolamine

LPG	Lysophosphatidylglycerol
LPI	Lysophosphatidylinositol
LPS	Lysophosphatidylserine
MAG	Monoacylglycerol
MGDG	Monogalactosyldiacylglycerol
OxPC	Oxidized phosphatidylcholine
OxPE	Oxidized phosphatidylethanolamine
OxPG	Oxidized phosphatidylglycerol
OxPI	Oxidized phosphatidylinositol
OxPS	Oxidized phosphatidylserine
PA	Phosphatidic acid
PC	Phosphatidylcholine
PE	Phosphatidylethanolamine
PEtOH	Phosphatidylethanol
PG	Phosphatidylglycerol
PI	Phosphatidylinositol
PMeOH	Phosphatidylmethanol
PS	Phosphatidylserine
SHexCer	SulfurHexosylceramide hydroxyfatty acid
SM	Sphingomyelin
SQDG	Sulfoquinovosyl diacylglycerol
TAG	triacylglycerol

Supplementary Figure

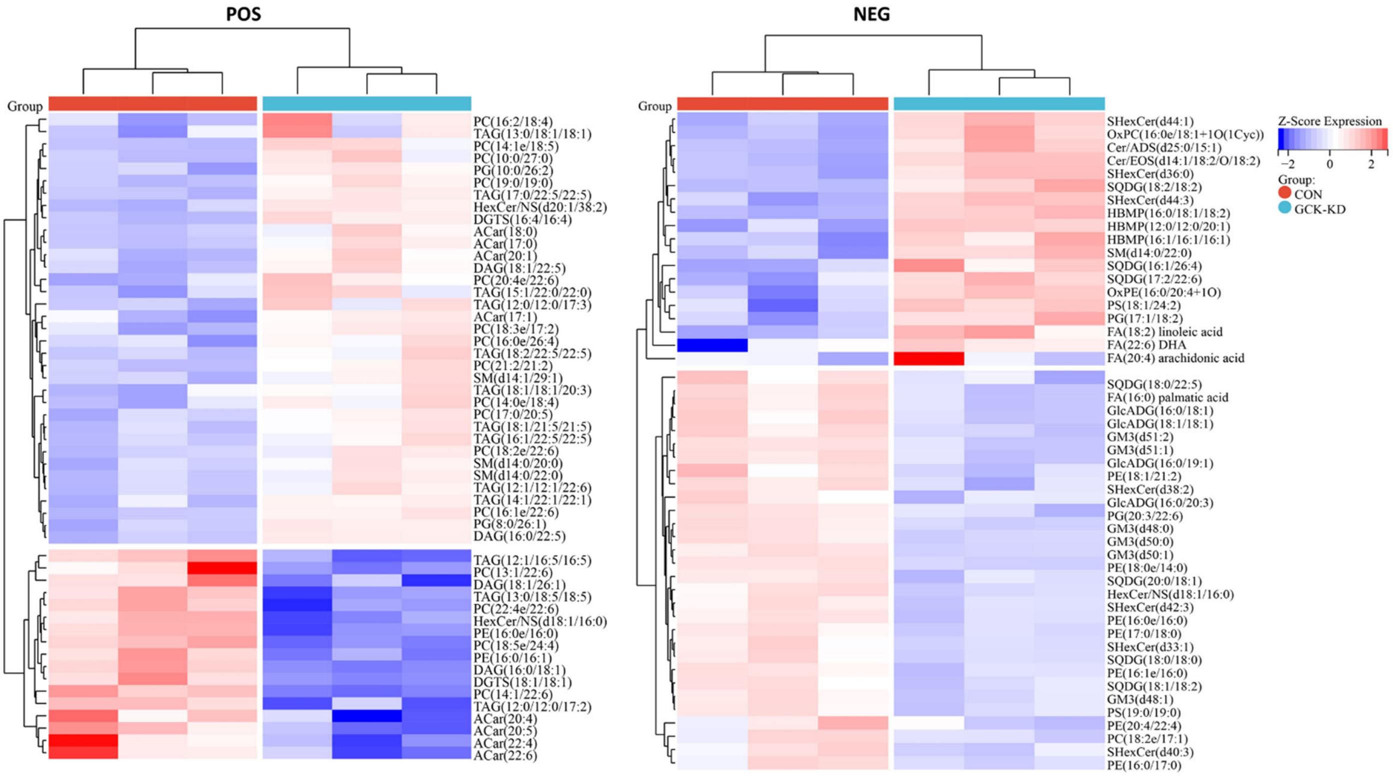

Figure S1. Heatmap of hierarchical clustering analysis for lipids in positive and negative mode. The color intensity presents the corresponding abundance difference. Blue indicates decreasing expression, and red indicates increasing expression. The heatmaps suggested that differential lipids could be clustered into two patterns in positive and negative modes, enriched in GCK-knockdown or control groups.

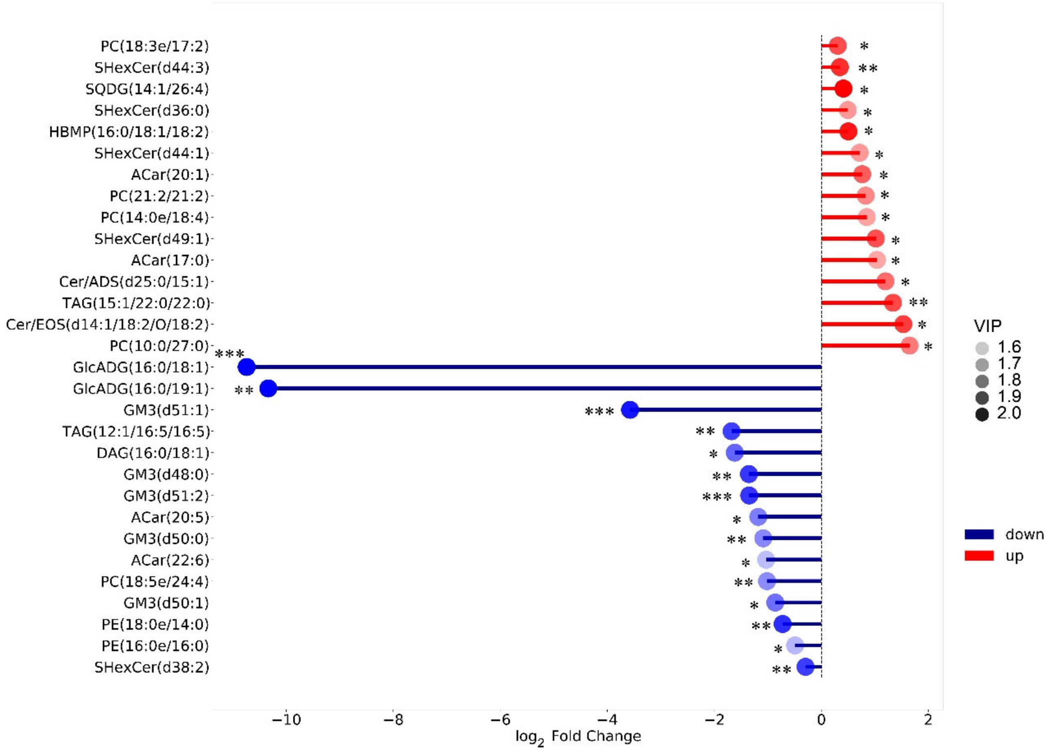

Figure S2. Matchstick analysis for differentially expressed lipids. The X-axis displays the log fold change of each lipid; the shade of dot color represents the VIP value. *** $p \leq 0.001$, ** $p \leq 0.01$, * $p \leq 0.05$ versus control group via Student's t-test.

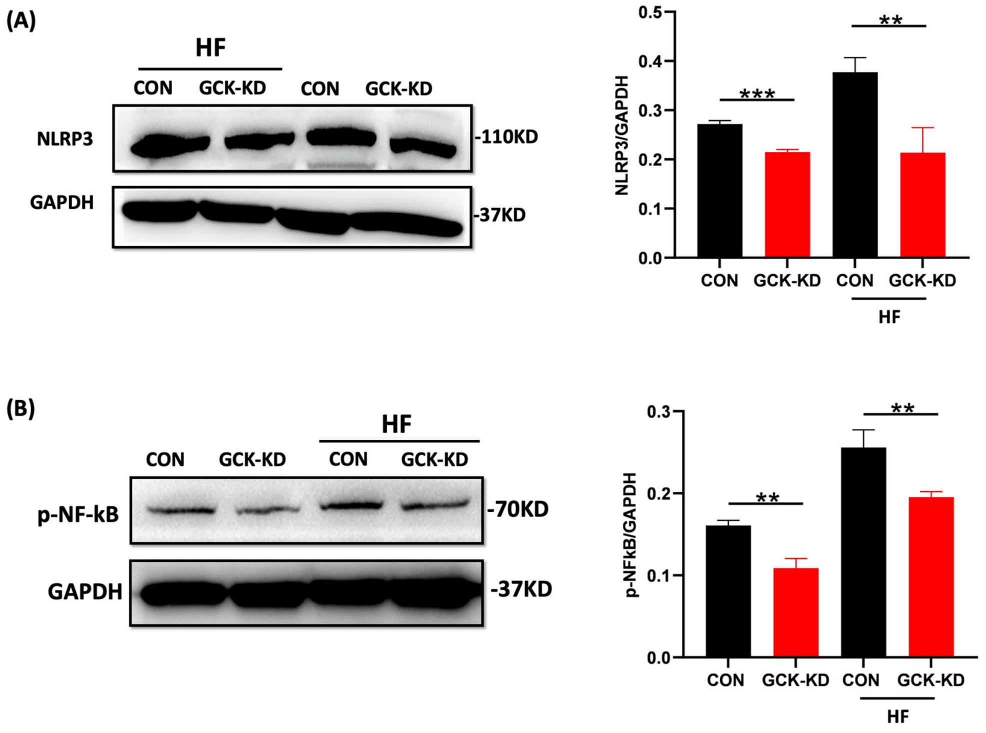

Figure S3. GCK knockdown impacts the expression of proteins involved in inflammation in HepG2 cells. (**A**) and (**B**) Relative expression of NLRP3 and p-NF-kB proteins in GCK-knockdown HepG2 cells under normal and high fatty acid (HFA) conditions. Data are represented as mean ± SD. *** $p \leq 0.001$, ** $p \leq 0.01$ versus control group via Student's *t*-test.
